# 525. Evaluating ChatGPT’s Potential as a Burn Evaluation Tool in Acute Care Settings

**DOI:** 10.1093/jbcr/irag033.222

**Published:** 2026-04-06

**Authors:** Francesco M Egro, Alexis M Henderson, Hilary Liu

**Affiliations:** University of Pittsburgh Medical Center, Pittsburgh, PA; University of Pittsburgh Medical Center, Mercy Burn Center, Pittsburgh, PA; University of Pittsburgh Medical Center, Pittsburgh, PA

## Abstract

**Introduction:**

ChatGPT is a widely accessible and versatile large language model. It has the potential to aid burn diagnosis by helping assess severity and guide initial care, particularly among first responders or underserved areas with limited access to specialists. Treatment depends on burn depth as a measure of injury severity. This study evaluates how well ChatGPT agrees with burn surgeons in identifying and managing burns to assess its potential in acute care.

**Methods:**

A dataset of 20 burn images, previously classified by a burn surgeon by depth, was used to survey other burn surgeons and ChatGPT on burn thickness, need for referral, and treatment approach. Inter-rater reliability was measured using Fleiss’s and Cohen’s Kappa and percent agreement, comparing surgeons’ responses, then surgeon consensus to ChatGPT’s responses.

**Results:**

There was 80% agreement among the three respondents (Fleiss’s kappa = 0.766 ± 0.046, *p*<.001), indicating substantial agreement. ChatGPT matched the consensus in 80% of cases (n = 48) with moderate but significant agreement (Cohen’s kappa = 0.413 ± 0.065, *p*<.001). Alignment was highest for deep partial (100%; n = 1) and full-thickness burns (86%; n = 6), and lowest for superficial burns (0%). For burn center referrals, agreement was 88% (n = 14) when indicated and 75% (n = 3) when not. ChatGPT and respondents fully agreed on conservative treatment (100%; n = 9) and showed 73% agreement (n = 8) for operative cases.

**Conclusions:**

ChatGPT aligned best with burn surgeons on clearly defined burn depths like deep partial and full thickness but struggled with superficial classifications. It performed well in identifying burns needing conservative treatment.

**Applicability of Research to Practice:**

ChatGPT’s strong agreement with burn surgeons supports its potential as a low-cost, accessible tool for burn evaluation in prehospital and acute care settings.

**Funding for the study: N/A**.

